# Noninvasive Surveillance and Evolutionary Insight into Siadenovirus among Antarctic Penguins

**DOI:** 10.1155/2023/9743267

**Published:** 2023-12-21

**Authors:** Rodrigo Arce, Irene Ferreiro, Joaquín Hurtado, Fabián Aldunate, Paula Perbolianachis, Diego Simón, Gonzalo Moratorio, Pilar Moreno, Juan Cristina

**Affiliations:** ^1^Laboratorio de Virología Molecular, Centro de Investigaciones Nucleares, Facultad de Ciencias, Universidad de la República, Igua, 4225, Montevideo 11400, Uruguay; ^2^Laboratorio de Evolución Experimental de Virus, Institut Pasteur de Montevideo, Mataojo, 2020, Montevideo 11400, Uruguay; ^3^Centro de Innovación en Vigilancia Epidemiológica, Institut Pasteur Montevideo, Mataojo, 2020, Montevideo 11400, Uruguay

## Abstract

Avian siadenoviruses infect diverse terrestrial and aquatic birds worldwide. Antarctica hosts several avian species that are susceptible to siadenovirus infection, such as penguins and South Polar skuas. However, the presence, diversity, and transmission of these viruses in Antarctic birds are poorly understood due to limited surveillance and sequence data. In this study, we performed a noninvasive surveillance of avian siadenoviruses using fecal samples collected from waterbirds at King George Island (part of South Shetland Islands, Antarctica) from late January to mid-February 2023. Polymerase chain reaction, sequencing, and phylogenetic analysis were used to investigate the occurrence, genetic diversity, and evolutionary relationships of these viruses in this unique environment. The results of these studies confirmed the presence of siadenoviruses in penguins living along the southeastern coast of King George Island. Distinct viral strains, specific to each penguin species studied, were found suggesting limited interspecies transmission and a complex viral ecosystem within Antarctic bird populations. Siadenovirus strains isolated from penguin's species were genetically distinct from those infecting South Polar skuas. An *in silico* 3D modeling of hexon proteins from siadenoviruses gathered from gentoo penguins permitted to detect key amino acid substitutions in the FG2 domain that may affect capsid structure and function. The persistent prevalence of siadenoviruses in Antarctica underscores the need for ongoing surveillance to understand the evolutionary dynamics of viruses in this region. This study is the first to noninvasively detect siadenoviruses in Antarctic penguins, opening a new avenue for viral research. This approach not only sheds light on viral dynamics but also contributes to the conservation of Antarctica's unique wildlife and biodiversity, especially in the face of increasing global warming.

## 1. Introduction

Adenoviruses are double-stranded nonenveloped, DNA viruses with a linear genome of approximately 25–45 kb [1, 2]. Adenoviruses have a similar structural morphology, but the terminal region of the genome differs between viruses of different genera [3]. Adenovirus virions contain three major capsid proteins, hexon (II), penton base (III), and fiber (IV); four minor proteins (IIIa, VI, VIII, and IX); and six other proteins (V, VII, *μ*, IVa2, terminal protein, and protease) [4]. The hexon is the largest and most abundant of the structural proteins of the characteristic icosahedral adenovirus capsid. The top of the hexon protein contains the loops (DE1, FG1, and FG2) and forms the outer surface of the viral capsid. The hexon base contains a small loop (DE2) and two eight-stranded “viral” jellyrolls (V1 and V2) separated by the connector (VC) [5].

The family *Adenoviridae* consists of six genera: genus Aviadenovirus, Atadenovirus, Ichtadenovirus, Mastadenovirus, Siadenovirus, and Testadenovirus [6]. Within genera, a species could be defined as having >10%–15% phylogenetic distance based on distance matrix analysis of DNA polymerase amino acid sequence or based on nucleotide composition [6].

Adenoviruses of wild birds are less well characterized than those of domesticated birds. However, these viruses can threaten not only their own host species but also other animals [7]. Most adenoviruses infect a single or narrow range of host species, but host switching has been observed [8].

Historically, all avian adenoviruses were classified in the genus Aviadenovirus, but based on genome organization and evolutionary studies, the genera Atadenovirus and Siadenovirus have been established for the divergent types [7].

Siadenoviruses have been detected in frogs, raptors, and turkeys [9–11], as well as in wild birds from various geographical regions of the world, including the Antarctic continent [12, 13]. The Antarctic region is highly isolated from the other continents. However, new microorganisms are introduced every year with the arrival of tourists and scientists to this little-explored continent with minimal exposure to human pollution [14, 15].

Siadenoviruses have been found to infect chinstrap (*Pygoscelis antarctica*) and gentoo penguins (*Pygoscelis papua*) [16], as well as south polar skuas (*Catharacta maccormicki*) [17]. Surveillance studies of adenovirus infection in Antarctic wild birds are limited and sequence data of siadenoviruses are currently scarce [7, 18]. In order to gain insight into adenovirus infection and evolution in Antarctic wildlife, we designed a surveillance strategy using feces as a noninvasive method approach. Samples were collected from aquatic birds in the coastal area of the Fildes Peninsula (King George Island) and polymerase chain reaction (PCR), sequencing and phylogenetic analysis of avian siadenoviruses were performed.

## 2. Materials and Methods

### 2.1. Sample Collection

**Figure 1:**
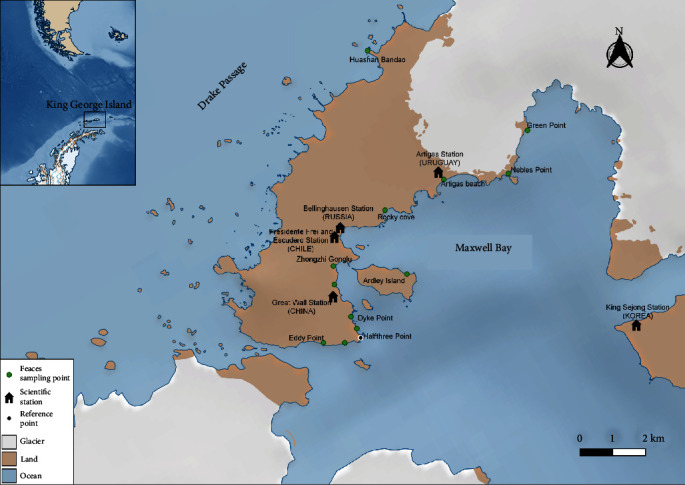
Sampling sites in Peninsula Fildes, King George Island, Antártica. Green dots indicate the locations where fecal samples were collected. Black dot indicates Half Three Point, a protected area in the peninsula, used as a reference point on the map. Black houses indicate the scientific stations located on the Fildes Peninsula. The map was created in QGIS3 (QGIS Development Team 2023), using the data package named Quantarctica [19].

Between late January and mid-February 2023, the coastal area of the Fildes Peninsula, King George Island, Antarctica (62°11′44′′S–62°12′ 40′′S and 58°54′67′′W–58°56′39′′W) was surveyed for fresh penguin feces (see Figure 1). To avoid invasive sampling techniques and disturbing wildlife, we waited at a considerable distance (5 m or more) from each penguin until they defecated. Once the penguins had moved away from the area, samples were collected. In addition to being noninvasive, this method ensures the collection of fresh fecal samples from live penguins and allows the correct registration of the species from which each sample was taken.

A total of 40 fecal samples were collected in sterile tubes, georeferenced, and stored at −20°C until processed. We were able to collect samples from gentoo (*Pygoscelis papua*) (*n* = 32), chinstrap (*Pygoscelis antarcticus*) (*n* = 7), and emperor (*Aptenodytes forsteri*) (*n* = 1) penguins.

### 2.2. DNA Extraction and PCR

Total DNA was extracted from a clarified 10% suspension in 1 × phosphate-buffered saline (PBS) by using the QIAamp DNA Mini Kit (QIAamp, USA). A clarified suspension was obtained by centrifugation at 6,000×*g* for 10 min and 200 *μ*L of the supernatant was used for the extraction according to the manufacturer's instructions.

Siadenovirus DNA was detected by PCR amplification of a 1200-bp region of the hexon gene. Q5 High-Fidelity 2X Master Mix (New England Biolabs, USA) was used according to the manufacturer's recommended protocol and under the following cycling conditions: an initial denaturation of 98°C for 30 s, followed by 35 cycles composed by denaturation at 98°C for 10 s, annealing at 58°C for 30 s, and extension at 72°C for 36 s, and a final extension for 5 min at 72°C, followed by a 10°C hold. Primers used in this PCR (Ad_hex1514F and Ad_hex2963R) were described by Lee et al. [16]. A gBlock synthetic DNA fragment [20] containing a 1,220 bp sequence segment of a previously reported Antarctic gentoo penguin siadenovirus full genome sequence (GenBank accession number: KP279747) was used as a PCR-positive control. A PCR fragment of 1,200 bp, corresponding to partial hexon gene sequences of Siadenovirus, was obtained.

### 2.3. Sequencing

PCR products were sequenced using the same sets of primers used for PCR amplification. Bidirectional Sanger sequencing was performed by Macrogen Korea (http://www.macrogen.com). The sequences of siadenovirus obtained in this work were deposited in Genbank under accession numbers OR513870 to OR513882.

### 2.4. Phylogenetic Analysis

The siadenoviruses sequences obtained from penguin fecal samples collected in Antarctica were aligned with siadenoviruses sequences isolated elsewhere, for which the complete genome sequences are known. Sequences were aligned using the MEGA 11 software [21]. For strain names, accession numbers, and hosts, see *Supplementary 1*. Once aligned, the best evolutionary model that described our sequence data was assessed using MEGA 11 program [21]. Akaike information criteria (AIC) and Bayesian information criteria (BIC) indicated that the HKY + *γ* + I model was an appropriate model to fit the sequence data (AIC of 8659.61 and BIC of 9063.64). Using this model, maximum-likelihood phylogenetic trees were constructed. As a statistical measure of the robustness of each node, we use the bootstrap method (1,000 pseudo replicas).

### 2.5. 3D Model of Siadenovirus Hexon

3D structures of siadenovirus hexon proteins are currently not available. In order to observe the positions of the amino acid substitutions found in the hexon protein of the Antarctic siadenovirus in a 3D structure, we modeled the complete hexon amino acid sequences of Chinstrap Penguin Adenovirus 2 (CSPAdV2) (GeneBank: KP144329) on the 3D hexon structure of CELO Adenovirus 2 (PDB: 2INY) using Phyre 2 software [22]. Ninety-nine percent of the hexon sequences from CSPadV2 were modeled with 100.0% confidence. As a measure of the reliability of the obtained model, a pairwise structural alignment was performed between the 3D structures of the obtained model and CELO Adenovirus 2 using the TM-align approach (available at https://www.rcsb.org/alignment). The TM score is a metric for assessing the topological similarity of protein structures, with values ranging from 0 to 1, where 1 indicates a perfect match between two structures. Structures with a score greater than 0.5 are generally assumed to have the same fold. A TM score of 0.97 was obtained for this comparison. Model coordinates can be found in *Supplementary 2*. All structures were visualized using the Jmol program (available at http://www.jmol.org).

## 3. Results

### 3.1. Siadenovirus Detection and Sequence Analysis

PCR studies showed that 14 out of 40 fecal samples collected (35%) tested positive for the presence of the siadenovirus genome. Of these 14 positive samples, 11 were isolated from gentoo penguins and three were from chinstrap penguins.

All the positive samples were collected along the Maxwell Bay coastline of the Fildes Peninsula, extending from Eddy Point to Nebles Point (see Table 1 and Figure 1 for geographic details). From these 14 positive samples, we obtained genomic sequences from 13. These sequences were deposited in Genbank under accession numbers OR513870 to OR513882. Unfortunately, we were unable to obtain the sequence of one of the gentoo penguin sample (GP_SAd6_KG).

### 3.2. Phylogenetic Analysis of the Hexon Gene from Siadenovirus Strains Isolated in Antarctica

**Figure 2:**
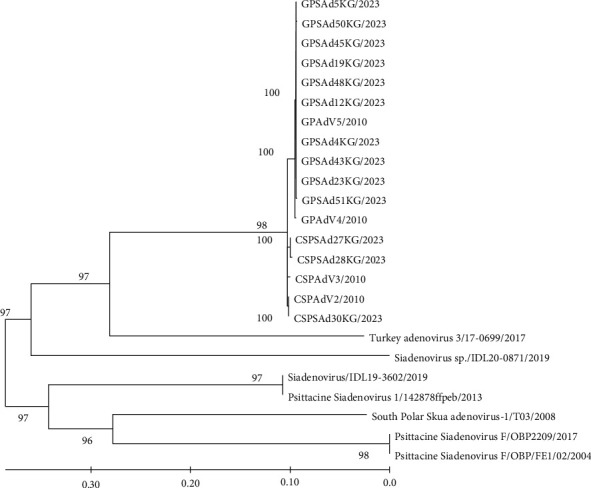
Maximum-likelihood phylogenetic tree analysis of hexon genes of siadenovirus. Strains in the tree are shown by name in bold for strains recently isolated from penguin species in Antarctica. Siadenovirus strains previously reported are shown by accession number followed by virus name. Numbers at the branches show bootstrap values. The bar at the bottom of the tree denotes distance.

In order to gain insight into the degree of genetic variability of Siadenovirus isolated in Antarctica, the 13 hexon gene sequences obtained from penguin's fecal samples in 2023 were aligned with corresponding sequences of 11 siadenoviruses isolated elsewhere, representing different avian siadenovirus species for whom the complete genome sequences are known. The analyzed region comprises 858 nucleotides of the hexon gene (positions 13,915–14,773) and includes the region encoding the V1, VC, V2, DE2, and FG2 domains of the adenovirus hexon protein (in relation to chinstrap penguin adenovirus 2 sequence, GeneBank: KP144329). Once aligned, phylogenetic trees were constructed using the maximum-likelihood method under the HKY + *γ* + I model. The robustness of the nodes was assessed using the bootstrap method (1,000 pseudo replicas). The results of these studies are shown in Figure 2.

All strains isolated from penguin samples in 2023 in Antarctica cluster together with strains isolated from chinstrap and gentoo penguin species, and each cluster was supported by very high bootstrap values. This finding suggests that strains isolated from penguin species have a closer genetic relationship with each other and a more distant genetic relationship with other siadenoviruses isolated elsewhere (see Figure 2). In fact, our results show that siadenovirus isolated from penguin species had 23% and 20% sequence differences with Turkey adenovirus 3 at nucleotide and amino acid levels, respectively, and 26%–29% and 23%–27% sequence differences at nucleotide and amino acid levels with Psittacine and other siadenoviruses. This indicates that siadenovirus isolated from penguin species represents a specific species in the Siadenovirus genera, in agreement with previous reports [16]. Interestingly, siadenoviruses isolated from penguin species have a distant genetic relationship with strains isolated from South Polar Skuas, with a nucleotide and amino acid sequence differences to strains isolated from penguin species of 28% and 24%, respectively. This indicates that at least two different siadenoviruses species are circulating in Antarctic wild birds, in agreement with previous reports [17].

### 3.3. Mapping of Amino Acid Substitutions in the Hexon Protein of Siadenoviruses

**Figure 3:**
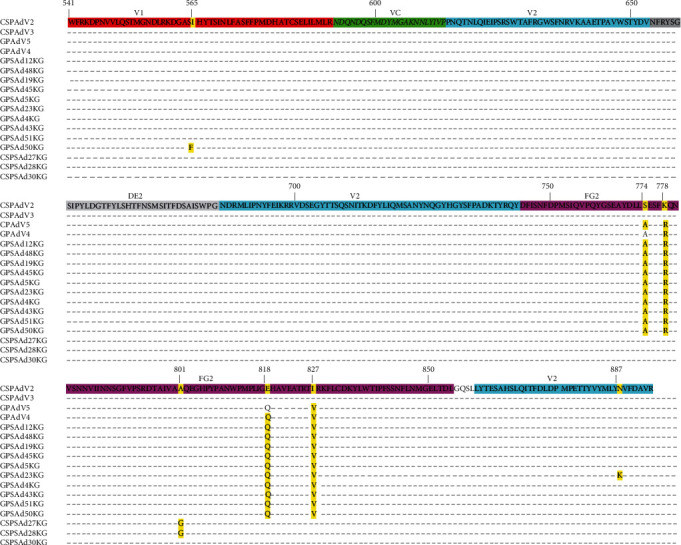
Amino acid substitutions found in the hexon protein of siadenovirus isolated in Antarctica. Partial amino acid sequence of the hexon protein from Chinstrap Penguin Adenovirus 2 (CSPAdV2) (GeneBank: KP144329) is shown. Domains of the adenovirus hexon protein are shown at the top of the amino acid sequence and in different colors. Substitutions with respect to CSPAdV2 are shown in yellow. Numbers at the top of the sequence denote amino acid position (in relation to CSPAdV2 sequence, GeneBank: KP144329).

To investigate whether the differences observed in the phylogenetic tree of the primary group of penguin siadenoviruses correlate with amino acid substitutions in the hexon protein, we performed an *in silico* translation of their sequences and aligned them to Chinstrap penguin adenovirus 2 (CSPAdV2, GeneBank: KP144329). The results of these studies are shown in Figure 3.

As viewed in Figure 3, several amino acid substitutions are observed in the FG2 domain of the adenovirus hexon protein between CSPAdV2 and 3 and siadenoviruses isolated from Gentoo penguins. Particularly, substitutions including S774A, K778R, E818Q, and I827V are observed in gentoo penguin adenovirus 4 and 5 (GPAdV4 and 5) in relation to CSPAdV2 and 3 (refer to Figure 3). The strains isolated in 2023 were found in the same area where both two penguin species coexist, suggesting that different adenovirus strains cocirculated simultaneously in penguin populations in Antarctica.

### 3.4. Mapping of Amino Acid Substitutions Found in the Hexon Protein of Siadenovirus Strains Isolated from Antarctica in a 3D Protein Model

**Figure 4:**
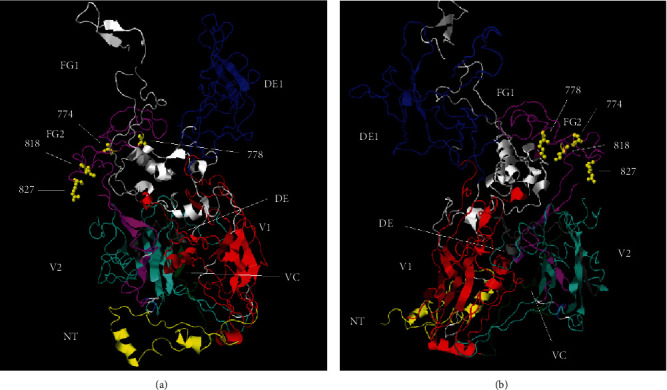
Hexon 3D model of Chinstrap Penguin Adenovirus 2 siadenovirus strain. Two views of the molecule rotated in the *x*-axis are shown in (a) and (b). Each domain of the protein is shown in different colors and their names are indicated next to the domain. Numbers indicate the positions of the amino acids where substitutions have been observed (according to CSPAdV2 sequence, GeneBank: KP144329).

To observe the amino acid substitutions found in this work in the FG2 domain of the hexon protein, we used a 3D protein model. Since there are currently no 3D structures available for hexon from siadenoviruses, we modeled the complete amino acid sequences of the CSPAdV2 hexon protein in the 3D structure of the CELO Adenovirus 2 hexon (PDB: 2INY). After obtaining the model, we performed a structural alignment between the model and CELO Adenovirus 2 hexon 3D structure. A TM score of 0.97 was achieved for the alignment, indicating a fitting model for analysis (refer to *Supplementary 2*).  Figure 4 displays the findings of these investigations.

The structural organization of the CELO hexon is similar to that of CSPAdV2 [5]. An extended loop on the basal surface of the protein, important for stability, is formed by the N-terminal region. This is followed by the first of two viral jellyrolls, V1. An extended and flexible structure is formed by the DE1 loop [5], which is then followed by the FG1 loop. The viral jellyroll connector domain (VC) is situated at the molecule's base, which separates the first (V1) and second (V2) viral jellyrolls clamps them together. The V2 domain, being the lesser of the two viral jellyrolls, possesses loop extensions between the DE and FG domains, but they are relatively smaller than the ones present in V1 domain. The DE2 loop is the smallest among the four hexon loops and performs the same function as VC in separating and stabilizing V1 and V2, but in the upper part of the jellyrolls. The FG2 loop, located at the top of the molecule, is significantly larger than the DE2 loop (refer to Figure 4). The FG2 domain substitutions identified in samples obtained from gentoo penguins, as well as in strains recently collected in Antarctica, are concentrated in the FG2 region that comprises the top of the hexon molecule (refer to Figure 4). This is in agreement with previous results revealing that although no conserved residues are distributed throughout the hexon structure, all of them are located on the molecular surface [5].

## 4. Discussion

The study of viral diversity within the Antarctic fauna is a vital endeavor, providing invaluable epidemiological and ecological insights into emerging pathogens in this isolated region [11]. While Antarctica's geographic and climatic conditions have historically kept it isolated, current evidence of climate change and animal behavior suggest potential pathways for the introduction and spread of infectious diseases [23]. Previous research has identified the presence of viruses in Antarctic avifauna, potentially transmitted by resident bird populations [24–27]. Recently, there have been reports of adenovirus infections in Antarctic avifauna, particularly in South Polar skuas (*Catharacta maccormicki*) and chinstrap penguins (*Pygoscelis antarcticus*), with these viruses belonging to the genus Siadenovirus [16, 17]. However, it is important to note that available sequence data for avian siadenoviruses remain limited [18].

To our knowledge, our study is the first to detect siadenovirus in noninvasively collected samples from live animals, which is in line with recent advances in viral detection techniques [7, 28–30]. A comparative analysis of our noninvasive sampling method with traditional invasive techniques, such as cloacal swabs in nearby areas, showed a similar positivity rate [16], confirming the efficacy of noninvasive methods for siadenovirus surveillance.

Previous studies carried out in Antarctica from 2008 to 2013 found a mean prevalence of 28.2% (22 positive cases among 78 penguins) for siadenoviruses in penguin populations [16]. In these studies, 40 fecal samples were collected from 40 different penguins (32 *Pygoscelis papua*, seven *Pygoscelis antarcticus*, and one *Aptenodytes forsteri*) in 2023, 14 of which resulted positive for siadenovirus (a prevalence of 35%) (see also Table 1). The results of this study suggest a continuous circulation over time of siadenovirus in penguin populations of Antarctica, as well as a significant prevalence of siadenovirus infection in these populations. A roughly similar prevalence was obtained in these studies by comparison with previous ones [16]. Although extreme care was taken in sample collection and no mixed sequences were obtained from positive samples, being this approach a noninvasive one that took fresh fecal samples, the possibility of an environmental contamination by a preexisting DNA, although low, cannot be ruled out.

In order to gain insight into the genetic variability and evolution of siadenoviruses in Antarctica, a phylogenetic analysis of recently isolated siadenoviruses from this region was performed. The results of these analyses showed that siadenoviruses isolated from Antarctic penguin species are closely related to each other and distantly related to other siadenoviruses isolated from other bird species (see Figure 2). Interestingly, siadenoviruses isolated from penguin species differ from siadenoviruses isolated from South Polar skuas (Figure 2). This is consistent with previous results showing that penguin adenoviruses are a new species within the genus Siadenovirus [16]. Furthermore, our analysis shows that siadenovirus partial hexon sequences effectively assign different siadenovirus species to appropriate clades, confirming previous studies using DNA polymerase partial gene sequences [16].

The results indicate that siadenoviruses found in chinstrap (*Pygoscelis antarctica*) and gentoo (*Pygoscelis papua*) penguins differ by at least four amino acid substitutions in the hexon protein (Figure 3). This suggests that there are at least two distinct penguin siadenovirus strains circulating at the same time in penguin populations, indicating genetic diversity among these strains. Further studies are needed to adequately address this important issue.

The adenovirus hexon protein is the major component of the viral icosahedral capsid. Previous research has identified common regions of hexon that are shared by different adenoviruses, whereas variable regions contribute to capsid diversity [5]. Currently, no 3D structure is available for the siadenovirus hexon protein. The hexon structure of chicken embryo lethal orphan (CELO) virus (PDB: 2INY) represents the only available structure of an avian adenovirus [31]. Previous research suggested that the overall structure of the CELO hexon closely resembles the hexons of human adenoviruses. The main differences between avian and human hexons are in the loops located at the top of the molecule [5]. To gain insight into this topic, a model of the full-length hexon sequences of chinstrap penguin adenovirus 2 (CSPAdV2, GeneBank: KP144329) was developed. The model is based on the 3D hexon structure of the CELO virus. The research uncovered a feasible model for CSPAdV2 hexon. The TM-align score between the CELO crystallographic hexon structure and the CSPAd2 model is 0.97 (refer to Figure 4 and *Supplementary 2*). Interestingly, amino acid substitutions located in the FG2 domain of the hexon protein of siadenovirus, isolated from gentoo penguin (*Pygoscelis papua*) (GPAd4 and 5) as well as recently isolated siadenovirus in Antarctica and CSPAdV2, are found at the top of the hexon molecule (see Figure 4). Further studies will be essential to assess the functional significance of these substitutions in the capsid conformation of siadenovirus strains prevalent in penguin populations in Antarctica.

## 5. Conclusions

In summary, our research demonstrates the presence of siadenovirus in penguins inhabiting the southeastern coast of the Fildes Peninsula.

Our results provide evidence for the distinct detection of viral strains within specific penguin species, suggesting limited interspecies transmission between gentoo and chinstrap penguins. Furthermore, our investigations reveal a remarkable genetic similarity among the siadenovirus strains detected in this study, which distinguishes them from siadenoviruses isolated from other hosts. In particular, the genetic dissimilarity between siadenoviruses found in penguin species and those isolated from South Polar skuas underscores the distinct evolutionary pathways of these viruses.

By constructing an *in silico* 3D model of the hexon protein of CSPAd2, we shed light on the spatial distribution of substitutions within the FG2 domain of siadenovirus hexon proteins isolated from gentoo penguins. These substitutions map predominantly to the uppermost region of the hexon protein, suggesting their potential importance in capsid conformation.

It is relevant to mention that the detection of siadenovirus in Antarctic penguins was performed using a noninvasive method. This issue not only advances our understanding of viral dynamics in this unique ecosystem but also plays a critical role in ongoing conservation efforts aimed at preserving Antarctica's wildlife and biodiversity.

The results obtained in this work highlight the ongoing circulation of siadenovirus within the Antarctic ecosystem and emphasize the need for continued vigilance in monitoring viral evolution in its fauna with noninvasive methods.

## Figures and Tables

**Table 1:** Collection sites of the positive siadenovirus samples.

Sample name	Accession number	Host species	Collection site coordinates	Fildes Peninsula region
GP_SAd4_KG	OR513876	Gentoo penguin	62°11′6′′S 58°52′42′′W	Nebles Point
GP_SAd5_KG	OR513870	Gentoo penguin	62°11′6′′S 58°52′42′′W	Nebles Point
GP_SAd6_KG	Not sequenced	Gentoo penguin	62°11′6′′S 58°52′42′′W	Nebles Point
GP_SAd12_KG	OR513875	Gentoo penguin	62°13′46′′S 58°57′5′′W	Between Eddy and Half Three Point
GP_SAd19_KG	OR513873	Gentoo penguin	62°11′40′′S 58°56′9′′W	Rocky Cove
GP_SAd23_KG	OR513878	Gentoo penguin	62°11′40′′S 58°56′9′′W	Rocky Cove
CSP_SAd27_KG	OR513880	Chinstrap penguin	62°12′50′′S 58°57′46′′W	Coast near Great Wall Station
CSP_SAd28_KG	OR513881	Chinstrap penguin	62°12′50′′S 58°57′46′′W	Coast near Great Wall Station
CSP_SAd30_KG	OR513882	Chinstrap penguin	62°12′31′′S 58°57′45′′W	Zhongzhi Gonglu
GP_SAd43_KG	OR513877	Gentoo penguin	62°12′38′′S 58°55′29′′W	Northeastern beach of Ardley Island
GP_SAd45_KG	OR513872	Gentoo penguin	62°12′41′′S 58°55′18′′W	Northeastern beach of Ardley Island
GP_SAd48_KG	OR513874	Gentoo penguin	62°12′41′′S 58°55′18′′W	Northeastern beach of Ardley Island
GP_SAd50_KG	OR513871	Gentoo penguin	62°11′7′′S 58°54′3′′W	Artigas beach
GP_SAd51_KG	OR513879	Gentoo penguin	62°11′7′′S 58°54′3′′W	Artigas beach

## Data Availability

The sequences were deposited in Genbank under accession numbers OR513870 to OR513882. The 3D model of the hexon obtained in these analyses is available in PDB format in *Supplementary 1*. Other data used in these analyses can be obtained from the corresponding author upon reasonable request.

## Supplementary Materials

Supplementary 1 Origins of the Siadenovirus strains.

Supplementary 2 Pairwise structure alignment of CELO and CSPAdV2 model hexon.
