# Supplementary material for: Noninvasive Surveillance and Evolutionary Insight into Siadenovirus among Antarctic Penguins
Source: Transbound Emerg Dis. 2023 Dec 21;2023:9743267. doi: 10.1155/2023/9743267 (PMC12017133; doi:10.1155/2023/9743267)
Supplement: Supplementary 1 — Origins of the Siadenovirus strains. [file 9743267.f1.docx]

**Supplementary Material Table 1. Origins of the Siadenovirus strains.**

______________________________________________________________________________________________________________

**Name Date of isolation Place of Isolation Host Accession number**

______________________________________________________________________________________________________________

Chinstrap Penguin Adenovirus 2 2010 King George Island *Pygoscelis antarctica* KP144329

Chinstrap Penguin Adenovirus 3 2010 “ “ KP144330

Gentoo Penguin Adenovirus 4 2010 “ *Pygoscelis papua* KP279746

Gentoo Penguin Adenovirus 5 2010 “ “ KP279747

GPSAd5KG 2023 King George Island “ OR513870

GPSAd50KG “ “ “ OR513871

GPSAd45KG “ “ “ OR513872

GPSAd19KG “ “ “ OR513873

GPSAd48KG “ “ “ OR513874

GPSAd12KG “ “ “ OR513875

GPSAd4KG “ “ “ OR513876

**Supplementary Material Table 1. Origins of the Siadenovirus strains. (Cont.).**

**______________________________________________________________________________________________________________**

**Name Date of isolation Place of Isolation Host Accession number**

**______________________________________________________________________________________________________________**

GPSAd43KG 2023 King George Island *Pygoscelis papua* OR513877

GPSAd23KG “ “ “ OR513878

GPSAd51KG “ “ “ OR513879

CSPSAd27KG “ “ *Pygoscelis antarctica* OR513880

CSPSAd28KG “ “ “ OR513881

CSPSAd30KG “ “ “ OR513882

Turkey adenovirus 3 2017 Canada *Meleagris gallopavo* MT603869

Siadenovirus sp. 2019 USA *Uria allge* MN480433

Siadenovirus IDL19-3602 2019 USA *Forpus coelestis* MK695679

Psittacine siadenovirus 1 2013 USA *Aratinga solstitialis* MN450070

Skua Adenovirus 1 2008 King George Island *Stercorarius maccormicki* HM585353

**Supplementary Material Table 1. Origins of the Siadenovirus strains. (Cont.).**

**______________________________________________________________________________________________________________**

**Name Date of isolation Place of Isolation Host Accession number**

**______________________________________________________________________________________________________________**

Psittacine siadenovirus F strain OBP2209 2017 Australia *Neophema chrysogaster* MW365934

Psittacine siadenovirus F strain OBP/FE1/02 2004 Australia “ OP377084

______________________________________________________________________________________________________________
