# Supplementary figures and images for: Noninvasive Surveillance and Evolutionary Insight into Siadenovirus among Antarctic Penguins
Source: Transbound Emerg Dis. 2023 Dec 21;2023:9743267. doi: 10.1155/2023/9743267 (PMC12017133; doi:10.1155/2023/9743267)

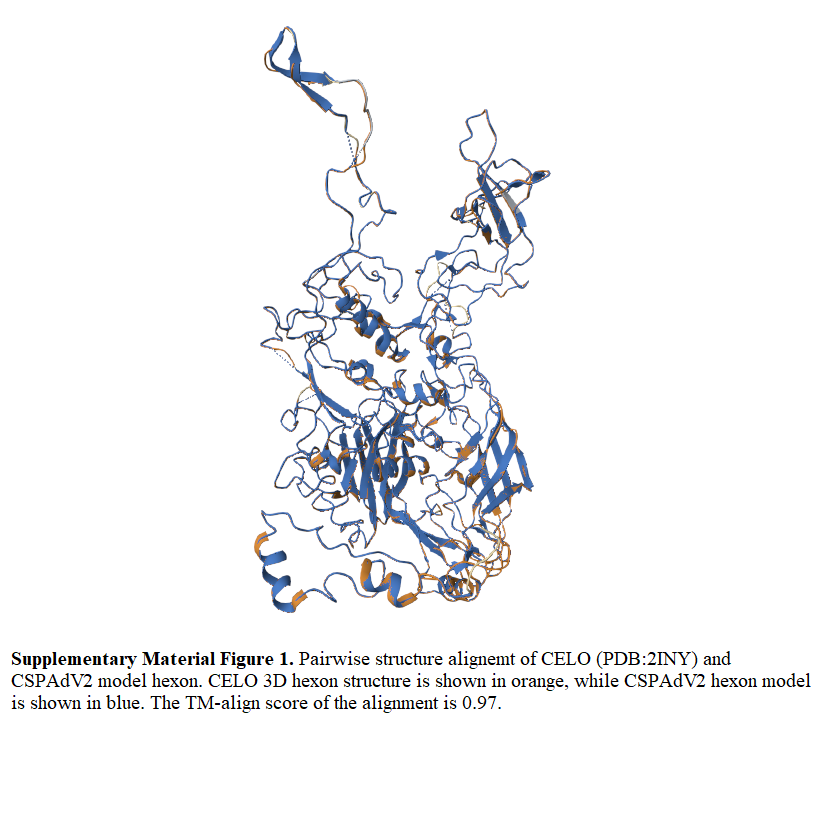

Supplement: Supplementary 2 — Pairwise structure alignment of CELO and CSPAdV2 model hexon. [file 9743267.f2.docx]
